# Case report: Minimally invasive removal of a dislodged thoracoamniotic shunt with an integral cystoscope in a preterm infant

**DOI:** 10.3389/fped.2023.1217667

**Published:** 2023-06-27

**Authors:** Lichao Zhu, Yanze Wang, Honghao Song, Xiaoqing Wang, Mingang Zhang, Feng Guo

**Affiliations:** ^1^ Department of Pediatric Surgery, Shandong Provincial Hospital, Shandong University, Jinan, China; ^2^ Department of Pediatric Surgery, Shandong Provincial Hospital Affiliated to Shandong First Medical University, Jinan, China

**Keywords:** fetal pleural effusion, thoracoamniotic shunt dislodgement, cystoscope, thoracoscopy, case report

## Abstract

**Introduction:**

Fetal pleural effusion is a rare condition that is associated with significant mortality. Although the insertion of fetal thoracoamniotic shunts can improve perinatal outcomes, there are several associated complications, such as intrathoracic dislodgement of the shunts. The optimal neonatal treatment for retained shunts remains uncertain.

**Case Description:**

A male infant was born at 32 weeks of gestation. He had antenatal hydrothorax that was detected at 27 weeks of gestation and was managed by intrauterine thoracoamniotic shunting. However, the shunt catheter dislodged into the fetal chest, which caused reaccumulated pleural effusion and respiratory distress requiring ventilatory support after birth. After the patient’s condition stabilized, minimally invasive removal of the retained catheter was performed on day 17 of life using an integral pediatric cystoscope via a 3-mm thoracic incision. The procedure took approximately 5 min. The postoperative course was uneventful, and the patient, who was discharged 39 days postnatally, is thriving at the 6-month follow-up.

**Conclusions:**

We present a novel and effective approach to the management of an intrathoracic shunt using an integral cystoscope. This approach may offer a valuable alternative to traditional thoracoscopy in the neonatal period.

## 1. Introduction

Fetal pleural effusion is a rare condition that occurs in 1 in 10,000-15,000 pregnancies. Massive pleural effusions can cause significant compression of the lungs and heart, leading to hemodynamic instability and nonimmune fetal hydrops, a potentially fatal condition if left untreated (1). Prenatal treatment typically involves the placement of fetal thoracoamniotic shunt(s) (TAS) that can significantly improve perinatal outcomes and survival rates in cases of large fetal pleural effusions (2–6). However, shunt dislodgement occurs in 5.4%–20% of cases (3, 6–9). Some previous studies suggested early elective removal of the shunt, primarily due to concerns regarding the risk of infection. In most cases, these shunts are extracted via thoracoscopy or a small skin incision (8, 10–12). Here, we detail our experience of removing a dislodged TAS from the chest using an “all in one” pediatric cystoscope via a 3-mm thoracic incision within 5 min.

## 2. Case description

**Figure 1:**
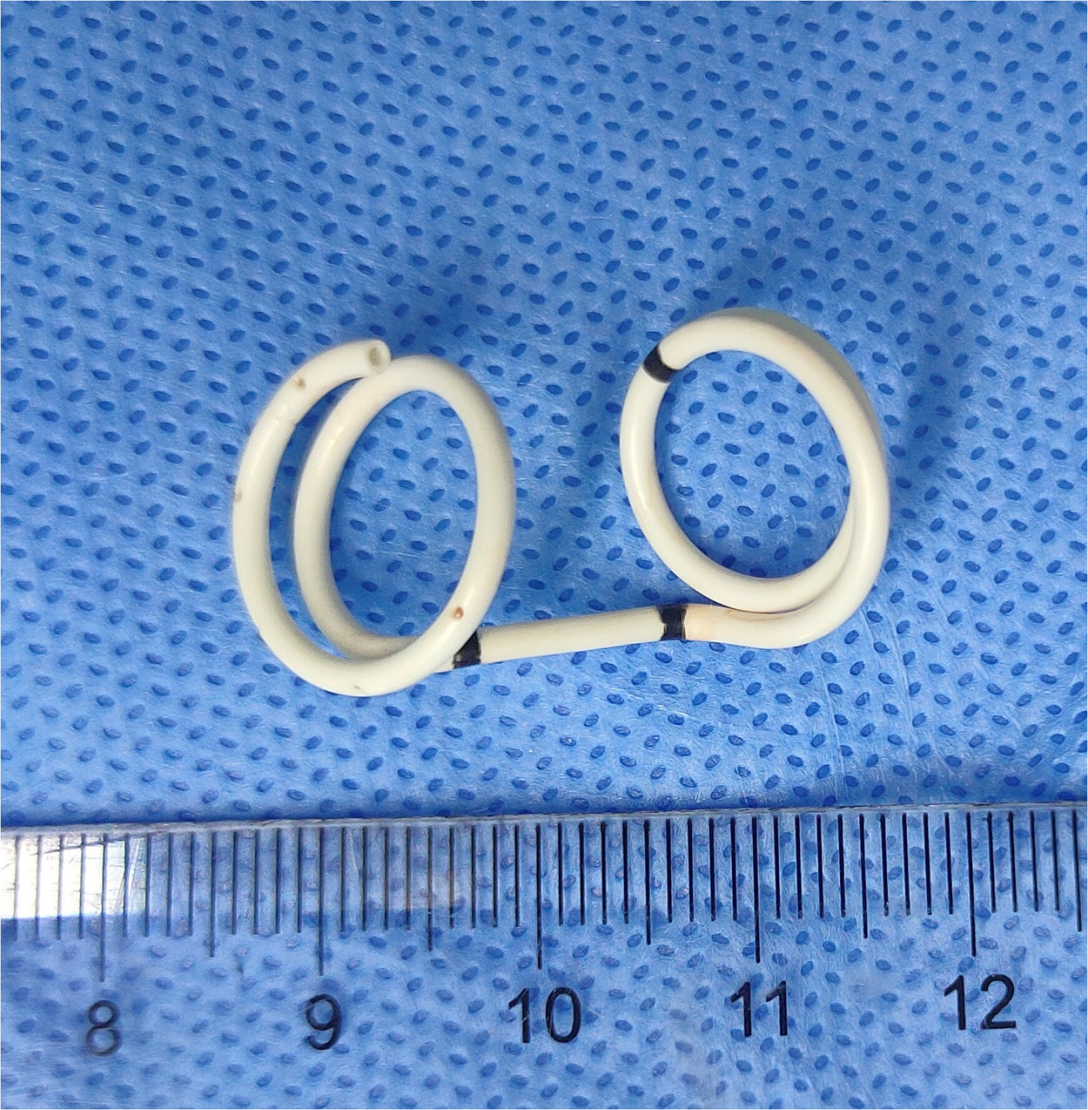
The fetal thoracoamniotic shunt is a double pigtail catheter (scale: centimeters).

**Figure 2:**
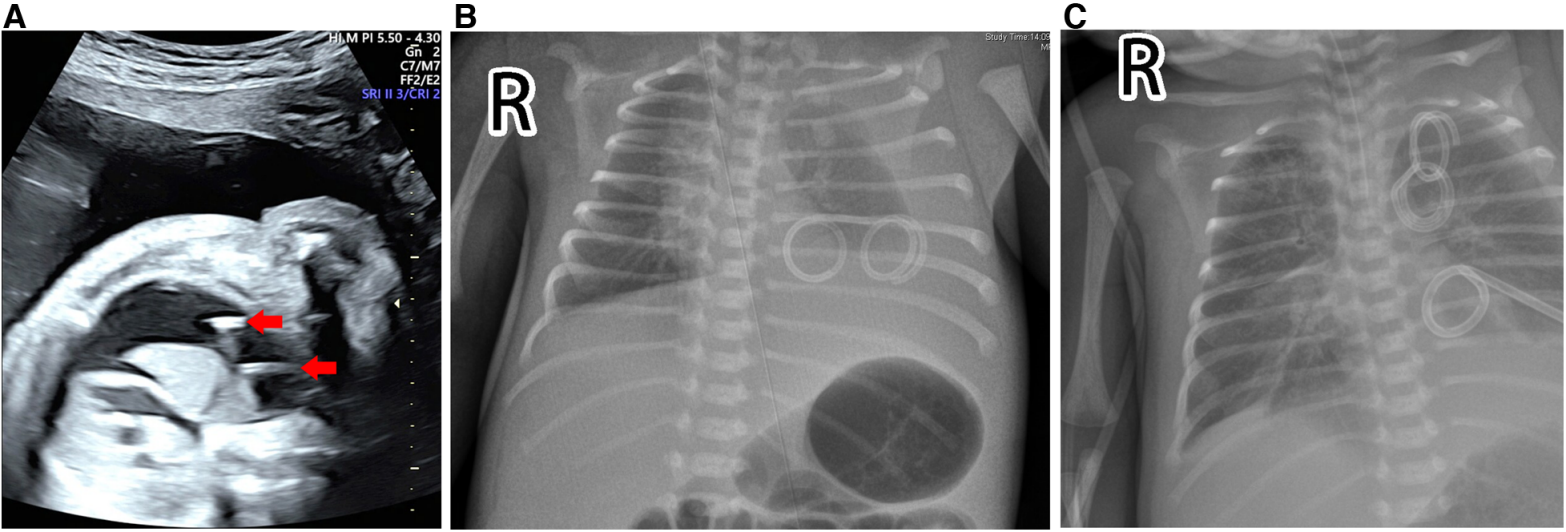
Prenatal ultrasound screening and postnatal chest radiography. (**A**) The ultrasound examination identified reaccumulated pleural effusion and clearly visualized the entire double-pigtail shunt (red arrows) located within the fetal chest, while the intra-amniotic segment remained invisible. (**B**) The chest x-ray displayed a massive left pleural effusion with dislodgement of the thoracoamniotic shunt. (**C**) The chest x-ray showed that the dislodged shunt had migrated to the left upper thorax.

A 31-year-old woman, gravida 5 para 3, was referred at 28 weeks of gestation after the detection of fetal pleural effusion during this pregnancy at 27 weeks. An ultrasound examination revealed left fetal pleural effusion with mediastinal shift to the right. Amniocentesis and ultrasound-guided intrauterine thoracocentesis were performed. However, the pleural effusion reaccumulated the following day. Under local anesthesia and ultrasound guidance, a TAS, which is a double-pigtail catheter, was placed without complications at 30 weeks of gestation (Figure 1). The ultrasound scan 24 h after the procedure showed resolution of both the pleural effusion and mediastinal shift. However, at 31^+2^ weeks, pleural effusion reaccumulated once again and this time, the whole TAS was clearly detected within the fetal chest but the intra-amniotic segment was invisible, indicating intrathoracic migration (Figure 2A).

At 32 weeks of gestation, a male infant weighing 2,300 g was delivered vaginally following spontaneous onset of labor. The infant’s respiratory condition was unstable, and chest x-ray revealed massive left pleural effusion with dislodgement of the thoracoamniotic shunt (Figure 2B). The preterm infant was intubated, and mechanical ventilation was started. Under ultrasound guidance, a 6 Fr pigtail chest tube was inserted into the left pleural cavity and initially drained 90 ml of pleural ﬂuid.

**Figure 3:**
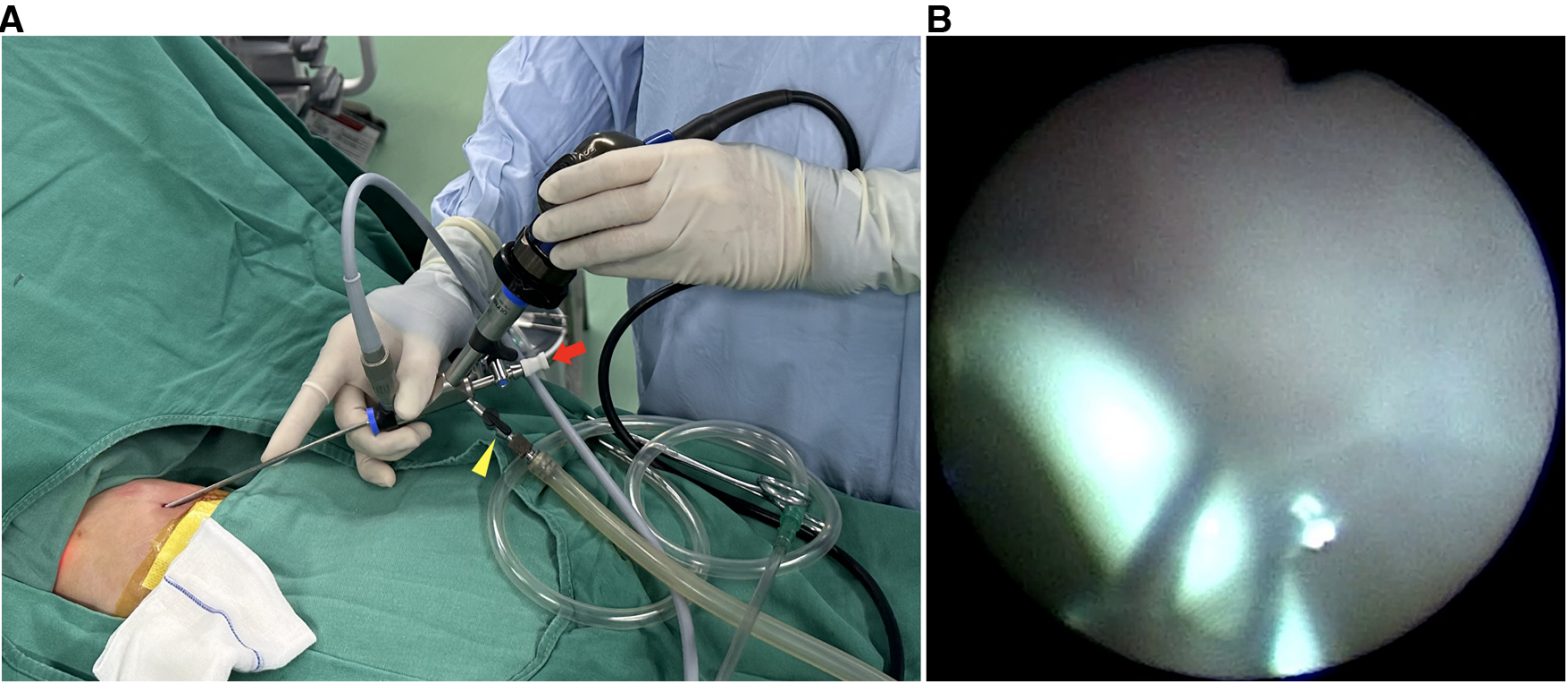
Operative images. (**A**) The cystoscope, which has a 4.2 Fr straight channel (red arrow) and two irrigation channels (yellow arrowhead), was inserted into the pleural cavity via a 3-mm incision without trocar insertion. (**B**) An endoscopic grasping forceps was inserted through the 4.2 Fr channel and grasped the shunt.

After evaluating the patient’s condition and the position of the shunt, which had migrated to the left upper thorax (Figure 2C), minimally invasive removal of the retained double-pigtail catheter was scheduled on day 17 postnatally. The patient was placed in the right lateral decubitus position, and general anesthesia was induced. A monitor was placed over the patient’s head. Instead of traditional thoracoscopy that typically requires optic and operating ports, we opted for an integral cystoscope (circumference 7.9 Fr, Compact Peadiatric Cystoscope, Olympus) with a united telescope and 4.2 Fr working channel to minimize the incision and reduce injury to the chest wall. A 3-mm incision was made overlying the fifth intercostal space in the mid-axillary line, and the cystoscope was inserted directly into the pleural cavity without trocar insertion (Figure 3A). Carbon dioxide insufflation of 4 mmHg was achieved through one irrigation channel of the cystoscope to improve visibility. However, massive pleural effusion and fibrous adhesions hampered identification of the TAS. We then gently performed blunt adhesiolysis using the rigid cystoscope as a thoracoscopic instrument and aspirated pleural effusion through another irrigation channel of the cystoscope. After careful inspection of the thoracic cavity, we located the shunt catheter behind the left upper lobe and extracted it using an endoscopic grasping forceps inserted through the 4.2 Fr working channel of the cystoscope (Figure 3B). The cystoscope was then removed, and a 10 Fr chest drainage tube was placed at the same incision. The operative time from insertion to removal of the cystoscope was approximately 5 min (Supplementary Video S1). The postoperative course was uneventful. The ultrasound scan confirmed no re-accumulation of the left pleural effusion, and the chest drains were removed on postoperative day 12. The patient, who was discharged 39 days postnatally, is thriving at the 6-month follow-up.

## 3. Discussion

Although the insertion of fetal TAS has been shown to improve fetal outcomes, there are several associated complications, including preterm birth, fetal constriction bands of the limbs, rib fractures, traumatic hemothorax, and shunt obstruction (3, 6, 9, 13, 14). Shunt dislodgement into the amniotic fluid, maternal peritoneal cavity, or fetal chest has also been reported in up to 20% of cases (3, 6–9). The mechanisms of shunt migration are not fully understood, but may include chest wall growth, respiratory movement, resolution of skin edema as hydrops improves, and technical difficulties encountered with hydropic fetuses or shunt insertion by less experienced operators (7, 15).

The management of shunts that have migrated into the thoracic cavity presents a dilemma, and the optimal neonatal treatment remains uncertain. Some studies have advocated for a conservative approach to the management of intrathoracic fetal chest shunts without surgical removal, as their follow-up did not reveal significant complications (9, 15). However, others have recommended early elective removal to minimize the risks of infection, the shunt’s proximity to mediastinal structures, and fibrous change that may complicate shunt removal (8, 10–12). There was a case reported by Blanch et al. where an abnormal position of a retained TAS led to strangulation of the pulmonary hilum, resulting in neonatal death (16). Therefore, we should be vigilant of the catastrophic consequences of dislodged shunts and recognize the need for early surgical intervention, especially when a retained shunt is located in the mediastinum near the lung hilum (12).

Regarding the ideal timing of intervention for intrathoracic dislodged shunts, most authors suggest removing them sooner rather than later, once the patient’s condition has stabilized (8, 10–12). A similar strategy was applied in our patient, in which the operation was performed on day 17 of life for initial stabilization. Compared with thoracotomy, thoracoscopic removal of retained TAS has several benefits, such as smaller incisions, clearer visualization of the thoracic cavity, less wound pain, fewer major wound complications, and faster recovery (11, 12). However, traditional thoracoscopy necessitates at least two incisions, measured 3–5 mm in length, for a camera port and a working port. Muta et al. reported their experience of removing TAS catheters with a 2.7-mm scope inserted into a 5-mm trocar to observe the thoracic cavity (10). Once the catheter was identified, a 3-mm forceps was inserted into the 5-mm trocar from the side of the scope to grasp the catheter. They believed that thoracoscopic removal with intraoperative radiography can help reduce their operation time, which averages 35.25 ± 30.49 min. Macchini et al. reported two newborns with intrathoracic dislodgement of TAS, where they inserted two 3-mm trocars and performed thoracoscopic removal of the shunts, with an operation time of 30 and 35 min, respectively (12). In our case, we confirmed the position of the dislodged TAS through preoperative x-ray. Intraoperative radiography is not necessary to perform routinely, as it may not provide more valuable information about the shunt location. We then chose an integral cystoscope, a one-piece instrument with a combined telescope, sheath, and working channels. The Olympus compact paediatric cystoscope (Product Number: A37026A), equipped with a 7-degree direction of view, a 4.2 Fr straight channel, and two irrigation channels, was the preferred choice. Its small outer diameter of 7.9 Fr makes one 3-mm incision adequate. Two irrigation channels can serve for carbon dioxide insufflation and pleural effusion suction. To locate the shunt, a rigid outer tube of the cystoscope can serve as a thoracoscopic instrument to lyse adhesions by the blunt technique under close monitoring. Once the shunt is identified, the 4.2 Fr straight channel of the cystoscope allows for the passage of an endoscopic forceps to grasp the catheter. An integral cystoscope is thus deemed valuable for removal of dislodged TAS. Additionally, pediatric rigid bronchoscopes, with diameters of 3–7 mm and lengths of 20–50 cm (17), may serve as a viable alternative for removing the dislodged TAS. However, at our institution, bronchoscopy is typically performed by pulmonologists and we do not have bronchoscopes readily available in the operating room. Therefore, we opted to use a cystoscope instead of a bronchoscope to remove the dislodged TAS.

As previously reported, severe intrathoracic adhesions can make it challenging to locate dislodged TAS in certain cases (10). Although we did not encounter this situation, the use of fluoroscopic radiography during surgery to detect the position of the TAS catheter could be beneficial, as described by Muta et al. These authors suggest that thoracoscopic removal with fluoroscopic radiography can help to reduce the operation time and wound length (10).

## 4. Conclusions

We present a novel approach to management of intrathoracic TAS with an integral cystoscope in the neonatal period. This procedure is safe, effective, and minimally invasive, and has the potential to improve clinical outcomes and reduce mortality. This integral cystoscope may offer a valuable alternative to traditional thoracoscope for patients with dislodged TAS.

## Data Availability

The original contributions presented in the study are included in the article/Supplementary Material, further inquiries can be directed to the corresponding author.

## Supplementary material

Video 1. An unedited video showing the entire procedure from the insertion to the removal of the cystoscope.
